# Mental health service users’ experiences of psychiatric re-hospitalisation - an explorative focus group study in six European countries

**DOI:** 10.1186/s12913-018-3317-1

**Published:** 2018-07-03

**Authors:** M. Ådnanes, L. Melby, J. Cresswell-Smith, H. Westerlund, L. Rabbi, M. Z. Dernovšek, L. Šprah, R. Sfetcu, C. Straßmayr, V. Donisi

**Affiliations:** 1 Department of Health Research, SINTEF Technology and Society, PO Box 4760, 7465 Trondheim, Norway; 2 Mental Health Unit, National Institute for Health and Welfare (THL), PL 30, 00271 Helsinki, Finland; 3 KBT Foundation (Competence center for experiential knowledge and service development), PO Box 934, 7409 Trondheim, Norway; 4 Department of Neurosciences, Biomedicine and Movement Sciences, Section of Psychiatry, University of Verona, P.le L.A. Scuro, 10, 37134 Verona, Italy; 5 Institute Karakter, Ježa 90, 1000 Ljubljana, Slovenia; 6 Research Centre of the Slovenian Academy of Sciences and Arts, Sociomedical Institute, Novi trg 2, 1001 Ljubljana, Slovenia; 7 National School of Public Health, Management and Professional Development, Bucharest (NSPHMPD), Faculty of Psychology and Educational Sciences, SHU Bucharest, Vaselor Street, No 31 Sector 2, 02125 Bucharest, Romania; 8 IMEHPS.research – Forschungsinstitut für Sozialpsychiatrie, Glasergasse 24/23, A-1090 Vienna, Austria

## Abstract

**Background:**

Psychiatric re-hospitalisation is considered costly and disruptive to individuals. The perspective of the mental health service user is largely unexplored in literature.

The purpose of our study was to explore service users’ experiences of psychiatric re-hospitalisation across six countries in Europe.

**Method:**

Eight focus groups were conducted in Romania, Slovenia, Finland, Italy, Austria and Norway.

**Results:**

A total of 55 service users participated in the study. All participants had been in receipt of mental health services for at least 1 year, and had experienced more than one psychiatric hospitalisation. The experience of re-hospitalisation was considered: (1) less traumatising than the first hospitalisation, (2) to be necessary, and a relief, (3) occurring by default and without progress, (4) part of the recovery process.

**Conclusions:**

Psychiatric re-hospitalisation was considered inevitable by the study participants, in both positive and negative terms. Striking similarities in service user experiences were found across all of the six countries, the first experience of psychiatric hospitalisation emerging as especially significant. Findings indicate the need for further action in order to develop more recovery and person-centred approaches within hospital care. For psychiatric inpatient care to be a positive part of the recovery process, further knowledge on what therapeutic action during the hospital stay would be beneficial, such as therapy, activities and integration with other services.

**Electronic supplementary material:**

The online version of this article (10.1186/s12913-018-3317-1) contains supplementary material, which is available to authorized users.

## Background

Psychiatric re-hospitalisation rate is a widely used quality indicator within mental health care. Reducing the number of psychiatric re-hospitalisations is a high priority in terms of health service efficiency and improving service user outcomes [1–3]. Data from Europe, USA and Canada indicate that up to 13% of mental health service users are re-hospitalised soon after discharge from acute psychiatric inpatient care [4]. Studies indicate that psychiatric hospitalisation and re-hospitalisations constitute profound interruptions to a person’s life, which may impact on quality of life. Exclusive use of hospital based care is considered inadequate and benefits of a pragmatic balance of community and hospital care is supported by evidence [5]. Furthermore, there have been reports of psychiatric inpatient wards being unsafe places to be [6, 7], may perpetuate social isolation, as well as and feelings of being dismissed, and not receiving adequate and appropriate treatment [7]. Despite these drawbacks, psychiatric hospital care may also be seen as a safe sanctuary of sorts, providing refuge and space to regroup and build resources [3]. This experience has predominantly been reported by involuntarily admitted patients, while others reported feelings of loss, fear and trauma [8].

Psychiatric re-hospitalisation is a complex and poorly understood phenomenon determined by a whole host of individual and system level factors. The need for a better understanding of this has become evident in the context of the EU-funded CEPHOS-LINK project “(https://www.thl.fi/fi/web/thlfi-en/research-and-expertwork/projects-and-programmes/comparative-effectiveness-research-on-psychiatric-hospitalisation)” including Finland, Norway, Italy, Slovenia, Austria and Romania. Through a series of literature reviews, the project explored a number of pre-discharge and post-discharge factors associated with psychiatric re-hospitalisation, both at individual and system level [9–12]. In line with service user experiences being increasingly recognised as an essential component in health services research, the CEPHOS-LINK project also explored experiences of psychiatric hospitalisation qualitatively. This is especially timely as capturing service users’ perspective on psychiatric re-hospitalisation remains largely unexplored [3, 13, 14]. Duhig and colleagues [3] used qualitative methods to study psychiatric re-hospitalisation from a service user perspective, describing it as a complex process, relating it to insufficiency of internal, interpersonal and/or environmental resources. The study was however limited to the Australian context. To the best of our knowledge, our study is the first to explore service user experiences of psychiatric re-hospitalisation across several European countries. The aim is to further our understanding of the multifaceted nature of psychiatric re-hospitalisation by including knowledge from service users’ experiences and perceptions.

## Method

Semi-structured focus groups were held in each participating country in order to elicit how service users regarded their experience of psychiatric re-hospitalisation.

### Study setting and sample

The mental health systems in the countries participating in our study differ a lot [15, 16]. Health insurance systems in Austria, Romania and Slovenia cover most types of mental health care, while Norway, Italy, Finland have tax funded systems. In Slovenia stand-alone psychiatric hospitals dominate, in Italy acute inpatient wards located in general hospital are the main stay; in the other four countries a mixture of types of hospital inpatient services exist. In Austria, Romania and Slovenia ambulatory care is mainly provided by insurance paid, office-based psychiatrists, while in the tax-based systems in Norway, Finland and Italy, psychiatric outpatient care is part of the public health system.

Participants who had been in receipt of mental health services for at least 1 year and experienced more than one psychiatric hospitalisation were invited to attend the focus groups. Participants were explicitly recruited via user organisations and/or activity−/day centres in each country. The recruitment inquiry was predominantly done by a dedicated contact person working in the organisation or centre. In some cases, a researcher visited the organisation providing information about the project and following up with invitations both verbally and in writing. Hence, a larger number of potential participants were received an invitation than those who actually participated in the end.

In Romania and Austria, participants were recruited from the capital cities. In Finland and Slovenia, the focus group was held in the capital city, but participants came from all over the country. In Norway focus groups were conducted in the third biggest city and in Italy in a city within the province of Verona.

### Data-collection

Focus groups were conducted between September 2016 and March 2017. Participants also completed a simple questionnaire collecting background information such as gender, age, education and housing, use of health services and diagnoses. Focus groups were conducted by a moderator (a researcher from the project group) and an assistant moderator (either another researcher or a professional from the service user organisation). A semi-structured interview guide was used to structure the focus groups (see Additional file 1: Interview guide). The interview guide was developed by the first author (MÅ) and the service-user representative and co-author of this article (HW). The focus group process was based on recommendations by Kitzinger [17], emphasising a good atmosphere, clearly explained aims, having an open introduction, well managed topics, attention to group dynamics, appropriate time allocation and following up the thematic guide with open questions.

The moderator’s task was to lead and maintain the focus of the interview, making sure all participants contributed, and encouraging discussion between participants. After initial introductions, the moderator opened the focus group discussion with a general question about the participants’ experiences of psychiatric hospitalisation and re-hospitalisation, including positive and negative experiences. Participation from all participants was ensured by turn taking.

The focus group interviews were held in local languages lasting between 60 and 90 min, were digitally recorded and transcribed verbatim. The interviews were then translated into English. Project researchers were responsible for conducting the focus groups, transcription and translation.

### Data-analysis

Transcripts of the focus groups were first read through in its entirety and then imported into HyperRESEARCH 3.7.3 qualitative software. Data was analysed based on principles of systematic text condensation [18]. The codes were created based on the data, first concentrating on the main themes in the interview namely, positive experiences with hospitalisation, negative experiences with hospitalisation, positive experiences with re-hospitalisation, negative experiences with re-hospitalisation. Codes were subsequently clustered to form descriptive themes, for example “lack of treatment during hospitalisation”. Descriptive themes that were related were further clustered together to form analytical themes, for example “the first admission was particularly traumatic”. All included themes were grounded in the text throughout the analysis.

Validity in qualitative research is based on “appropriateness” of the tools, processes, and data [19]. To increase the credibility and trustworthiness, the data-analysis was performed separately by two researchers (in Norway). Transcripts from all countries were read by a smaller group of researchers in the team. Agreement on all analytical themes was reached via a series of internal meetings discussing data and analyses. Transcripts were also read by several of the focus group participants (in Austria and Finland). Additionally, a representative from a Norwegian service-user organisation was actively involved in the entire research process, evaluating the interview-guide, participating as a co-moderator and discussing the results with researchers as well as co-authoring the article.

## Results

The research sample included material from a total of 55 service users who had experienced more than one psychiatric hospitalization (see Table 1). A total of eight focus groups were held, consisting of one or two groups in each of the participating countries. Each focus group consisted of six to nine participants.

**Table 1:** Number of focus groups and participants in each country

Country	Number of focus groups	Number of participants
Romania	1	8
Austria	2	12
Slovenia	2	14
Finland	1	6
Italy	1	9
Norway	1	6
Total	8	55

It was found that 36.4% (*n* = 20) of the participants had been admitted to psychiatric hospital during the last twelve months, while 50.9% (*n* = 28) had been admitted during the last 3 years (see Additional file 2: Table S1). For the rest of the participants (*n* = 7), more than 3 years had lapsed since the last psychiatric hospital stay. Most of the participants reported several psychiatric diagnoses; 41.8% (*n* = 23) with psychotic disorders, 38.2% (*n* = 21) with bipolar disorder, 21.8% (*n* = 12) with a depressive disorder and 12.7% (n = 7) with anxiety disorder. During the last year 63.6% (*n* = 35) had had regular outpatient visits to psychologist or psychiatrist, 56.3% (*n* = 31) had had contact with their general practitioner and 21.8% (*n* = 12) had visited a psychotherapist. A total of 25.5% (*n* = 14) had received rehabilitation services and 18.2% (*n* = 10) had visited a day hospital.

The majority of the participants were female 60.0% (*n* = 33) their ages ranging from 26 to 65 years. More than a quarter, 25, 5% (*n* = 14) had completed undergraduate or postgraduate degrees at university or college, and 10.9% (*n* = 6) had attended university or college without completing a degree. A total of 38.2% (*n* = 21) had completed secondary/high school as their highest level of education and 25.5% (*n* = 14) had completed only primary school. 45.5% (*n* = 25) lived alone.

Analysis identified four distinct themes: (1) re-hospitalisation as less traumatising than the first hospitalisation (2) re-hospitalisation as a necessity and a relief, (3) re-hospitalisation by default without progress, (4) re-hospitalisation as part of the recovery-process.

Quotes from the focus groups are coded to denote country and number of focus group (for countries which had more than one focus group) as well as participant number.

### Theme 1: Re-hospitalisation as less traumatising than the first hospitalisation

Participants did not describe re-hospitalisation in the same dramatic terms as experiences surrounding their first hospitalisation. The first hospitalisation was described by many as something shocking, intolerable, and terrible. The majority of the participants across all six countries described their psychological distress also in relation to the onset of a severe mental disorders, several of them using words such as “trauma” and “traumatising” to describe the experience of being hospitalised. Illustrated in the word, of the following participant:

“For me, my first admission…it was… (..). It was a trauma, a very big one. (..) I knew what I wanted, I was fit to plead, but it was something “induced” by someone else.” (I/7).

Several participants experienced particularly stressful situations in relation to being involuntarily admitted. The results show negative effects in conjunction with the admission, but also in longer terms, as described by the following participant:

“I must say that now - the second time around was better, although the hospitalisation itself, the way they held me in was terrible. I must say that I was still scared because of it for a while. One day I was at a shopping mall, after I was released from the hospital, when I came home. There was a group of people, and I was immediately afraid that they will attack me. That was because I was tied down when I was admitted to the hospital - they came from behind me. So, I needed a quite some time to realize these things. That these are just normal people. That this has nothing to do with the events there [in the Hospital].” (S1/3).

Psychiatric re-hospitalisation on the other hand encompassed a degree of familiarity. Several participants reported that they had a better idea of what to expect, perhaps already familiar with members of staff which contributed to a greater sense of security as described below:

“The only thing that I can say in this regard, there is a difference if you have seen the personnel and the doctors already. You know them and are known; well, that makes a bit of a difference. [...] [If you] at a re-hospitalisation get into a setting that you already know—okay; with him or her you have to behave like this and the people also know, with him/her you have to behave a bit like that, that can sometimes also make it a bit more pleasant.” (A1/6).

Although participants also viewed this familiarity as a negative thing, some for example describing how difficult it was to return to a place where staff may already hold a particular (at times erroneous) opinion about the service user. One participant expressed this as follows:

“So, concerning re-admission, I only was in there [Hospital] twice, but I encountered the attitude a bit, of myself and also of the personnel, at the first time I said, “see you, never again”, they all just looked foolishly and thought, well, we’ll probably see her again soon […] the second time I heard it [“see you, never again”, from the Hospital personnel], and it was rather well-meant, but it actually also expresses an attitude.” (A2/4).

### Theme 2: Re-hospitalisation as necessary and a relief

Re-hospitalisation was considered by many participants across all six countries as necessary at times, for example during crisis. Some participants described how they had postponed seeking psychiatric hospital care hoping it would not be necessary, while others talked about not being able to obtain a referral for psychiatric hospital care even though they strongly felt that it was necessary. In this context, hospitalisation could potentially be associated with feelings of relief in terms of finally getting the help they wanted, as described by the following:

“I felt relieved, because I had battled for a long time to get [hospital] care.” (F/5).

Relief was also expressed among participants who had been involuntary admitted, some describing the paradox of going through a difficult time and experiencing feelings of relief at the same time, for example:

“I’m so into my own world that I do not understand that I’m sick. [..] They did what they had to do. I stopped taking my medicine, so they made me take it again, and it went well.” (N/2).

Others described re-hospitalisation as providing some respite, a form of structure and a temporary release from responsibilities:

“Yes, it was a relief that I went, like I was going to a vacation. So, I can rest my brain and pull myself together and then forward again.” (S2/14).

“It is positive, that there I can withdraw completely from all the people who are getting on my nerves in the crisis, from housekeeping and work and responsibility, which certainly has already become too much for me, otherwise I wouldn’t be in hospital.” (A2/6).

This theme also included whether participants perceived their mental health difficulties as a long term, with some participants acknowledging and that they would sometimes need to be re-hospitalised to ease periods of crisis, even though it involved both positive and negative experiences. As one participant stated: “Relapses are always around the corner” (I/3).

### Theme 3: Re-hospitalisation as inevitable and by default but without progress

Some participants were highly critical of the mental health system itself, perceiving psychiatric re-hospitalisation as something inevitable or happening by default while offering no support or progress or recovery. While theme 2 reflected the acceptance and acknowledgement of periods of psychiatric hospitalisation despite the negative connotations, theme 3 reflected a more critical stance. A few participants even blamed mental health services themselves of sustaining mental health problems, illustrated by the following:

“[..] now it’s like people come [to hospital] again and again, and why do people come in again and again and again? Well, people come in again and again and again because psychiatry “chronicises” instead of helping people regain their health.” (F/2).

Consistent with this view, another criticism was directed at hospital assessment practices, viewing re-hospitalisations as something that happened “by default”:

“I mean, they do not evaluate you, whether you were… hospitalised five times in Hospital x. They do not care anymore about the current state. The present state is simply put described by default.” (R/1).

Participants not only talked about a lacking specialised mental health services, but some also pointing out a lack of adequate services in the community resulting in repeated hospitalisations.

### Theme 4: Re-hospitalisation as part of a recovery-process?

Quite another perspective, expressed in different ways by a few participants, was accepting psychiatric re- hospitalisation as a normal part of life within the recovery process. This illustrated by the following participant who compared psychiatric with somatic re-hospitalisation:

“In my opinion, you have to think that it [re-hospitalisation] is a part of life. Admission to hospital is natural; it is not a big deal. If you have to go to the hospital to have your appendix removed, not many people question that, right? But, if you have [a chronic illness], it is partly a defeat, but partly you should also think that it is natural.” (N/1).

This perspective assumes the possibility of an active inpatient-stay. However, satisfaction in the amount of activity and therapy offered during the hospital stay varied among the participants. For some, having an adequately structured day was deemed satisfactory, while others expressed disappointment and voiced critical views in terms of the hospital’s ability to actually cure them:

“Spontaneously, it occurs to me that psychiatry does not heal you. You don’t go to hospital and are discharged healthy. This is simply not the case.” (A1/1).

Many participants across all six countries mentioned the lack of therapy and activities as a problem, leading to too much time being spent on their own:

“There is occupational therapy and there is occupational therapy, but all that only here a bit and there a bit and all there was of occupation I soaked it all in. But that was simply not enough for me.” (A1/2).

There was an expectation, especially in relation to the first hospitalisation that one would receive treatment during the hospital stay. When no treatment was provided, participants reported being left on their own:

“You are by yourself too much [in the hospital]. Maybe some psychotherapy would be good [..]. That would be necessary to implement. I think there is not enough of it. It is a positive thing though, that we have art activities.” (S1/1).

## Discussion

### Summary of findings

This study depicts the experiences of psychiatric re-hospitalisation among mental health service users from six European countries. Following qualitative thematic analyses, participants’ perceptions of re-hospitalisation were found to vary a great deal, centering around four key themes. Theme 1 indicates that re-hospitalisation appears to be far less traumatic than first experiences of psychiatric hospitalisation pointing to certain advantages of being a more “experienced” patient. Theme 2 reflects a self-reported need for re-hospitalisation, with participants acknowledging a need for hospital care, despite both positive and negative previous experiences. Feelings of relief were also connected to this theme seeing inpatient care as means to obtain necessary help. Theme 3 examined system-level criticism citing psychiatric re-hospitalisation as something inevitable which happens by default, without resulting in progress or recovery. Theme 4 sees psychiatric re-hospitalisation as part of the recovery process, making comparisons to inpatient care for somatic illness.

Commonalities of experiences of re-hospitalisation emerged across all six countries involved in the study.

### Comparison with wider literature

Our study suggests that negative experiences associated with the first psychiatric hospitalisation remains significant even after many years have passed. Allowing patients the opportunity to discuss their experiences and address potentially traumatic experiences appears to be of value here. Lessons learned during the first psychiatric hospitalisation appear to contribute to subsequent experiences of re-hospitalisations with participants more familiar around what to expect, having already developed coping strategies from previous stays. Previous studies indicate that traumatic experiences during the first psychiatric hospitalisation are negatively related to willingness to engage in future treatment [20], however a more recent study only found modest evidence of this [21].

Our second theme described feelings of relief associated with (re)hospitalisation has also been a topic of discussion in several previous studies [3, 22]. In fact, our European study had several similarities with Duhig and collegues’ [3] Australian study. The Australian study also described feelings of relief and sanctuary as well as viewing re-hospitalisation as a default mechanism within the mental health care system. Psychiatric re-hospitalisation occurring by default is supported also by previous research, which also highlights previous hospitalisations being consistently the most significant predictor of re-hospitalisations [1]. Duhig and collegues describe psychiatric re-hospitalisation not as an event, but as part of a complex process including respite from both individual coping mechanisms and stress and struggle in the community. Duhig et al. underline psychiatric re-hospitalisation as the “tip of the iceberg” acknowledging that many problematic issues remaining unrecognised or unseen. Seeing psychiatric re-hospitalisation as a process and not as an event supports a more recovery-oriented approach.

Different definitions and models view recovery as an ongoing process toward improvement in health and quality of life rather than a set of interventions with a distinct endpoint [23]. For healthcare services to be recovery oriented, Duhig and colleagues suggested that stronger integration of health and social services is needed and steering the focus away from conventional interventions which focus on changing individuals. A Norwegian study [24] make similar conclusions highlighting the need for more integrated services and pointing out that identification of service users’ own goals is central to the recovery process. The Norwegian study also highlights service users’ expectations of treatment was to be able to understand and cope with ones’ mental health difficulties but also to receive support in terms of social life, family issues, work, education and financial issues, all considered vital for recovery [24].

One perspective within theme 4 of our study sought to normalise long term mental health difficulties in line with somatic illness within society. Recovery literature emphasises first and foremost what is possible by supporting the individual outside of the hospital. The prospect of an inpatient-stay as part of the recovery process is more seldom discussed. A recent study of recovery-focus in acute inpatient settings in England and Wales concluded that despite ideas of recovery being evident, there was some uncertainty about the relevance of recovery based ideals in inpatient care, including questions around the ability of people in acute distress to engage in recovery-focused approaches [25]. There is however consensus that good quality care should be made available to mental health service users in order to promote recovery both in an inpatient context or within the community [26]. Self-referral to hospital treatment is an example of how the recovery model could be used in a hospital based context, the main point being that service-users themselves determine their need for inpatient care without referral from health professionals. This also supports the service-user to seek help at an early stage reducing the probability of acute or involuntary admission at a later stage [27].

The nature of the hospital stay could be part of the patient’s recovery process, and a step towards wiping out the distinction between the hospital and other services. This perspective, however, demands a much more active in-patient-stay than participants in our study experienced, both in terms of therapy and activities. Participants in our study mentioned the need for more therapeutic activities during hospital stays. This has been highlighted also in previous studies with Thomas and colleagues [22] investigating what is considered therapeutic within an inpatient facility. Thomas et al. concluding that while refuge from self-destructiveness is essential, there was a wish for a deeper connection with staff, as well as access to more intensive insight-oriented therapies. Similarly, a review by Hopkins et al. indicates that service-users expect hospital stays to include developing positive relationships with nurses and other staff members in a safe environment [28]. This finding echoed by Gilburt and colleagues [13] stating that that relationships formed the core of service users’ experiences, considered essential for hospitalisation to achieve a positive impact.

### Strengths and limitations

The primary strength of this study is that it covers experiences from a broad range of mental health service users with different psychiatric diagnoses across several countries representing different traditions and healthcare systems in Europe.

The validity of the data was safeguarded in several ways, adding further value to the study. The study design was implemented in the same way in all six countries, by experienced researchers. Transcripts were read by several of the focus group participants, and a service user-representative participated in the entire research process. The analyses were performed in a multi-professional manner including input from a a psychologist and a sociologist, incorporating interpretations from different perspectives.

The study sample was equally distributed across different age groups, with the exception of age groups below 26 and above 65 years of age. More women than men participated (60% versus 40%) in the focus groups.

Including participants who felt comfortable in discussing the research was crucial for this study, however could have contributed to a degree of selection bias by naturally including participants with a higher level of education and social functioning. This effect has also been discussed in other qualitative studies [29], recognising that service users’ who are more motivated or engaged with mental health services may be more likely to participate in research studies.. It is however also possible that some participants may have held back on sensitive information in the interviews, although the richness of the data suggests that this was unlikely.

Although the limited number of participants from each country prohibited a thorough analysis of country differences, the explorative design of the study allowed for important reflections on commonalities and differences. It was noteworthy for example, that although the CEPHOS LINK countries were purposefully selected due to their heterogeneity, it appears that attitudes and experiences expressed in our qualitative study were remarkably consistent, independent of the context.

It is noteworthy that the selection of focus group sites in three of the countries was guided by the location of the local research groups, meaning that results from those countries may reflect specific characteristics of inpatient services available in those regions. Moreover, differences between countries may emerge as a bigger issue in future studies focusing in more detail on participants’ opinions of alternatives to psychiatric hospitalisations in terms of services, measures and local resources.

## Conclusion

Our qualitative study shows that service users considered psychiatric hospitalisation to be inevitable from time to time. An important distinction emerged in terms of re-hospitalisation as necessary, as a relief and for their own benefit versus occurring by default, being forced upon them by the healthcare system without resulting in progress or recovery. Striking similarities in attitudes towards, and experiences of psychiatric re-hospitalisation emerged across all six countries involved in the study.

The strong criticism and ambivalence that many of the participants expressed, suggests that mental healthcare systems still have a long way to go in terms of responding to the service users’ individual needs. There is clearly a need for healthcare services to develop more person-centred approaches and follow-up practices, also in the context of inpatient treatment. The current study highlights the first psychiatric hospitalisation as particularly important in this context.

In order for psychiatric inpatient treatment to be a positive step in the process of recovery, considerably more therapeutic action is needed during hospital stays including therapy, activities and improved integration with other services. Reducing distinctions between hospital and other mental health services may contribute towards improved continuity of care.

## Additional files

Additional file 1: (Interview guide) (DOCX 16 kb)

Additional file 2: (Table S1) (DOCX 19 kb)

## Abbreviations

CEPHOS-LINK: Comparative effectiveness research on psychiatric hospitalisation by record linkage of large administrative data sets
