# Supplementary material for: Mental health service users’ experiences of psychiatric re-hospitalisation - an explorative focus group study in six European countries
Source: BMC Health Serv Res. 2018 Jul 3;18:516. doi: 10.1186/s12913-018-3317-1 (PMC6029175; doi:10.1186/s12913-018-3317-1)
Supplement: Supplementary file 1 — (Interview guide) (DOCX 16 kb) [file 12913_2018_3317_MOESM1_ESM.docx]

**INTERVIEW GUIDE**

1. How does it feel like to be hospitalised within mental health care?

• First "brainstorm" in your group - what is positive and negative aspects related to a hospitalisation (first regardless of whether it is a readmission or not).

a. Checking out positive factors:

• Feeling relief

• Need Treatment

• Need rest and tranquillity - releasing responsibility

• Security

• Have expectations about getting treatment

• etc

b. Checking out negative factors:

• Insufficient treatment - more "storage"

• Is partially or completely taken out of daily life - work, school etc

• It takes long time

• Feels like defeat

• Cases of coercion (hospitalization, medication, restricted freedom)

• There are too undifferentiated in relation to individual needs (eg norm of time)

• etc.

2. How does it feel to have one or more readmissions - also, compared to the first admission (try to reveal factors that come in addition to the above)?

• Distinguish between different circumstances (if relevant):

• Acute

• Scheduled

• User-driven - eg based on 'Coping/ crisis Plan' or other tools

• Any other types

- Were there any alternatives to readmission as you see it?
- What kind of alternatives (to readmission) are there *in general* where you live?

3. What factors may be relevant/important in terms of *avoiding* readmission?

- Factors related to services and supply and availability of these:

• What happens before readmission

• The continuity of care and follow up/treatment in general when hospitalized

• Preparing for discharge (while hospitalized)

• Prematurely discharged / long inlaid

• Follow up in the municipality after discharge:

- Treatment, work; housing, day centers / activities etc.

• Preventing readmission by short-term option:

- Partial hospitalization "intensive" outpatient, social housing,

• Preventing readmission by means of more permanent option:

- ACT etc.
- Factors related to the person him/herself:

• Stability / instability in life

• Importance of social networks and support from family / friends

• Impact of substance abuse and other diseases

• The degree of self-mastery / setting

• Past experience

4. Is an inpatient stay an experience you are open about, or do you prefer not to share it with other people (felt as stigmatizing)?
