# Supplementary material for: Mental health service users’ experiences of psychiatric re-hospitalisation - an explorative focus group study in six European countries
Source: BMC Health Serv Res. 2018 Jul 3;18:516. doi: 10.1186/s12913-018-3317-1 (PMC6029175; doi:10.1186/s12913-018-3317-1)
Supplement: Supplementary file 2 — (Table S1) (DOCX 19 kb) [file 12913_2018_3317_MOESM2_ESM.docx]

**Table S1: Description of the participants**

| **Category** | **Variable** | **Per cent (n)**  **N** |
| --- | --- | --- |
| Sex | Male | 40.0 (22) |
|  | Female | 60.0 (33) |
| Age (years) | 26–35 | 22.2 (12) |
|  | 36–45 | 24.1 (13) |
|  | 46–55 | 29.6 (16) |
|  | 56–65 | 24.1 (13) |
| Highest education | Completed primary school | 25.5 (14) |
|  | Completed secondary/high school | 38.2 (21) |
|  | Exams from College/University (without degree) | 10.9 (6) |
|  | Completed lower degree at University/College | 9.1 (5) |
|  | Completed higher degree at University/College | 16.4 (9) |
| Living situation | Living with parents | 12.7 (7) |
|  | Living with husband/wife, partner | 14.5 (8) |
|  | Living with sister or brother | 3.6 (2) |
|  | Living alone with own child(ren) | 7.3 (4) |
|  | Living with relatives | 1.8 (1) |
|  | Living with other co-habitants | 3.6 (2) |
|  | Living with other co-habitants in residential care | 10.9 (6) |
|  | Living alone | 45.5 (25) |
| Psychiatric diagnoses (multiple entries possible) | Psychotic disorder | 41.8 (23) |
|  | Depressive disorder | 21.8 (12) |
|  | Bipolar disorder | 38.2 (21) |
|  | Anxiety disorder | 9.5 (7) |
|  | Other | 14.9 (11) |
